# Aloperine Ameliorates Acetaminophen-Induced Acute Liver Injury through HMGB1/TLR4/NF-*κ*B and NLRP3/Inflammasome Pathway

**DOI:** 10.1155/2024/3938136

**Published:** 2024-10-01

**Authors:** Hui Chen, Shu Wang, Qiuyue Chen, Wen Yu, Hao Nie, Lian Liu, Bing Zheng, Quan Gong

**Affiliations:** ^1^ Department of Laboratory Medicine The First Affiliated Hospital of Yangtze University, Jingzhou, China; ^2^ Department of Immunology School of Medicine Yangtze University, Jingzhou, China; ^3^ Hubei College of Chinese Medicine, Jingzhou, China; ^4^ Clinical Molecular Immunology Center School of Medicine Yangtze University, Jingzhou, China; ^5^ Department of Pharmacology School of Medicine Yangtze University, Jingzhou, China

## Abstract

**Purpose:**

Aloperine (ALO), an alkaloid isolated from *Sophora alopecuroides* L., possesses multiple pharmacological activities and holds a promise potential for the treatment of various clinical conditions, including skin hypersensitivity, cancer, and inflammatory disorders. The purpose of this study was to investigate the role of ALO in acetaminophen (N-acetyl-para-aminophenol (APAP))-induced acute liver injury and its underlying mechanisms.

**Materials and Methods:**

An animal model of acute liver injury was induced by intraperitoneal injection of APAP (150 mg/kg). Prior to APAP injection, ALO (40 mg/kg) was administered daily for 7 consecutive days. Serum alanine aminotransferase, aspartate aminotransferase, and lactate dehydrogenase levels were then measured using an automated chemical analyzer. Histopathological changes were evaluated using hematoxylin and eosin staining. Oxidative stress levels were measured by detecting superoxide dismutase (SOD), glutathione (GSH), and malondialdehyde (MDA). Pro-inflammatory cytokines were detected in serum and liver tissues using ELISA and quantitative real-time polymerase chain reaction (q-PCR). The expression of members of the HMGB1/TLR4/NF-*κ*B signaling pathway and NLRP3 inflammasome were determined by Western blot and/or q-PCR. In addition, the expression and location of NLRP3, cleaved caspase-1, high-mobility group box 1 (HMGB1), and phosphorylated p65 (p-p65) were detected by immunofluorescence.

**Results:**

Pretreatment with ALO significantly protected mice from APAP-induced acute liver injury, with decreased MDA content, and significantly increased GSH and SOD activities. Furthermore, ALO pretreatment reduced the release of pro-inflammatory cytokines (IL-1*β* and TNF-*α*) and decreased the expression of caspase-1, cleaved caspase-1, and NLRP3. In addition, ALO pretreatment also inhibited the activation of the HMGB1/TLR4/NF-*κ*B signaling pathway.

**Conclusion:**

Taken together, ALO can ameliorate APAP-induced acute liver injury by inhibiting oxidative stress, inflammation by inhibiting the HMGB1/TLR4/NF-*κ*B, and NLRP3/inflammasome pathway.

## 1. Introduction

Acute liver injury (ALI) is a severe liver disease that can be caused by drug-induced toxicity, viral infection, excessive alcohol intake, autoimmune liver disease, and radiation damage [1]. The incidence of ALI has been on the rise in recent years, a trend that correlates with the increased use of herbal products and dietary supplements. ALI is typically characterized by an acute inflammatory response and hepatocyte necrosis, which can significantly impair liver function. If not addressed promptly, ALI may progress to a range of complications, and in severe or persistent cases, it can escalate to acute liver failure (ALF). Currently, strategies for enhancing liver function encompass the use of trypsin inhibitors, agents that promote liver regeneration, and subcutaneous liver tissue engineering [2]. While these treatments have shown promise primarily in laboratory settings, translating their success from animal models to clinical practice presents numerous challenges [3]. Therefore, it is imperative to develop safe and efficacious therapeutic agents that can ameliorate ALI.

N-acetyl-para-aminophenol (APAP), also known as acetaminophen, is an antipyretic and analgesic drug that is commonly used in clinical practice. It is generally considered to be safe and effective when used at recommended doses. However, APAP overdose can easily lead to ALI, and even ALF and death in severe cases [4]. Although researchers are currently developing new drugs for the treatment of APAP-induced ALI [5, 6], all of these studies are still in the experimental or “bench” phase. At present, N-acetylcysteine (NAC) is considered to be the most effective treatment for APAP-induced ALI in clinical practice [7]. This is due to NAC's ability to replenish glutathione (GSH), a critical antioxidant in the liver that is depleted during APAP overdose. However, NAC has a narrow therapeutic window, most patients have missed the best time for treatment before admission to the hospital, resulting in a high incidence of acute liver disease. It has been reported that the incidence of APAP-induced liver failure in Western countries is 35%–70% [8, 9]. Therefore, it is necessary to develop new drugs to alleviate drug-induced liver injury (DILI), thereby reducing the risk of severe liver failure in patients.

During an APAP overdose, APAP can be metabolized into a hepatotoxic product, N-acetyl-p-benzo-quinone imine (NAPQI), which can be detoxified by GSH. However, long-term overdose of APAP can lead to depletion of GSH, accumulation of NAPQI, mitochondrial dysfunction, and the production of large amounts of reactive oxygen species (ROS), resulting in oxidative stress and the activation of inflammation cascade therefore inducing ALI [10, 11]. High mobility group box 1/toll-like receptor-4 (HMGB1/TLR4) is a classic inflammatory signaling pathway that promotes the release of inflammatory cytokines (TNF-*α*, IL-1*β*, and IL-6) by activating downstream nuclear factor-kappa B (NF-*κ*B) [12]. In addition, inhibition of TLR4/NF-*κ*B signaling can block the activation of the inflammasome NLRP3, thereby ameliorating APAP-induced liver injury [13]. Some studies have shown that mice lacking NLRP3 or caspase-1 were less prone to APAP-induced ALI [14, 15]. Similarly, NLRP3 or caspase-1 inhibitors can also alleviate APAP-induced ALI [16, 17]. Therefore, inflammasome pathway is involved in the pathogenesis of APAP-induced ALI.

Aloperine (ALO), an alkaloid extracted from *Sophora alopecuroides* L., has shown various biological effects on atherosclerosis [18], allergic asthma [19], liver fibrosis [20], liver cancer [21], and rectal cancer [22]. In addition, ALO also exerts good effects on some inflammatory diseases. For example, ALO can suppress allergic airway inflammation in the ovalbumin (OVA)-induced asthmatic mouse model by regulating NF-*κ*B signaling pathways [19]. Furthermore, ALO suppresses lipopolysaccharide (LPS)-induced inflammatory responses in RAW264.7 macrophages via inhibition the secretion of pro-inflammatory cytokines, TNF-*α*, IL-6, and IL-17A [23]. In addition, ALO improves the dextran sodium sulfate-induced colitis by inhibiting the PI3K/Akt/mTOR signaling [24]. However, the effect of ALO on ALI remains unclear. Therefore, the present study aimed to observe whether ALO has a protective role for APAP-induced ALI; in addition, the expression of signaling pathways such as HMGB1/TLR4/NF-*κ*B and NLRP3/inflammasome was analyzed to elucidate the possible mechanisms involved.

## 2. Materials and Methods

### 2.1. Animals

Specific pathogen free, male BALB/c mice (8 weeks; 23–25 g) were provided by the Center for Animal Experiments of China, Three Gorges University (Yichang, China). All mice were raised for 1 week at 24 ± 2°C in specific pathogen-free conditions, with a 12 hr light/dark cycle prior to the experiment. All experimental procedures were in accordance with the Guide for the Care and Use of Laboratory Animals (National Institutes of Health, Bethesda, MD, USA) and were approved by the Animal Ethics Committee of Yangtze University (Jingzhou, China).

### 2.2. APAP-Induced ALI and Experimental Design

Mice were randomly divided into four groups including vehicle, ALO, APAP, and ALO/APAP groups. APAP (Sigma, St. Louis, USA) was dissolved in pyrogen-free phosphate-buffered saline (PBS) that had been preheated to 40°C. The ALO, with a purity of ≥98% (Kmaels, Shanghai, China), was dissolved in PBS containing 10% acetic acid at a concentration of 2.5 mg/ml. Before the APAP treatment, mice in the ALO and ALO/APAP groups were administered ALO (40 mg/kg/day) via gavage for 7 consecutive days [24]. Concurrently, the vehicle and APAP groups were treated with an equal volume of PBS containing 10% acetic acid. One hour after the final gavage, mice in the APAP and ALO/APAP groups were intraperitoneally injected with APAP (150 mg/kg), while mice in the vehicle and ALO groups received an equal volume of PBS. Twelve hours after the APAP injection, all mice were anesthetized with ether [25]. Subsequently, blood was collected via retro-orbital bleeding, and the mice were then euthanized by cervical dislocation. Liver samples were collected and a portion of each sample was processed for pathological analysis, which included routine dehydration, fixation, and embedding to create tissue sections. The remaining liver tissue was stored at −80°C for further analysis.

### 2.3. Assessment of Liver Function

Blood samples were collected in 1.5 ml Eppendorf tubes without heparin and left on ice for 2 hr. Serum was collected after centrifugation at 3,600 rpm for 15 min at 4°C. The degree of liver injury was assessed by measuring serum alanine aminotransferase (ALT), aspartate aminotransferase (AST), and lactate dehydrogenase (LDH) levels using an automated chemical analyzer (Beckman, USA) according to standard procedures.

### 2.4. Histopathological Examination

A sample of the right lobe of the liver was collected and fixed with 4% neutral formaldehyde for 48 hr. Then, liver samples were dehydrated in a series of gradient ethanol concentrations, cleared in xylene, embedded in paraffin, and sliced into 5 *μ*m thick sections. After sections were deparaffinized, standard hematoxylin–eosin (H&E) staining was performed, and histopathological lesions were observed by light microscopy (Leica, Germany).

### 2.5. Detection of Oxidative Stress Indicators

Liver tissue, stored at −80°C, was homogenized in a 10% (w/v) solution of ice-cold 0.05 M potassium phosphate buffer with a pH of 7.4. Following homogenization, the mixture was centrifuged at 4,000 rpm for 15 min at 4°C. The resulting supernatant was collected and set aside for subsequent experiments. The activities of hepatic glutathione (GSH), superoxide dismutase (SOD), and malondialdehyde (MDA) in the supernatant were determined using commercial assay kits, strictly according to the manufacturer's instructions (Jiancheng Institute of Biotechnology, Nanjing, China).

### 2.6. Evaluation of the Level of Inflammatory Cytokines

The levels of inflammatory factors IL-1*β* and TNF-*α* in serum were measured using commercial ELISA kits (MultiSciences, Hangzhou, China). All steps were performed according to the manufacturer's procedure.

### 2.7. Quantitative Real-Time PCR Analysis (q-PCR)

Total RNA from liver tissue was extracted using the TRIzol reagent (Ambion Life, Technologies, Carlsbad, CA, USA) following the manufacturer's protocol. The extracted RNA was then reverse-transcribed into complementary DNA (cDNA) using a PrimeScript RT reagent kit (Takara, Shiga, Japan). Quantitative real-time PCR (q-PCR) analysis was conducted on the Ambion 7500 real-time PCR system, employing SYBR Premix Ex Taq™ kit (TaKaRa, Shiga, Japan) for amplification. The relative expression levels of the target genes were determined using the 2^−*ΔΔ*Ct^ methods [26], with glyceraldehyde-3-phosphate dehydrogenase (GAPDH) gene serving as the endogenous control for normalization. The primers for q-PCR were designed using Primer 5.0, and the sequences were as follows:

HMGB1 forward 5′-GAT GGG CAA AGG AGA TCC TAA G-3′, reverse 5′-TCA CTT TTT GTC TCC CCT TTG GG-3′

TNF-*α* forward 5′-CTT GCC CTC TAC AAC CAA CA-3′, reverse 5′-ACT TGC GAC CCA CGT AGT AGA-3′

IL-1*β* forward 5′-CAT CCA GCT TCA AAT CTC GCA G-3′, reverse 5′-CAC ACA CCA GCA GGT TAT CAT C-3′

NLRP3 forward 5′-TAC GGC CGT CTA CGT CTT CT-3′, reverse 5′-CGC AGA TCA CAC TCC TCA AA-3′

GAPDH forward 5′-GGT TAT CTC CTG CGA CTT CA-3′, reverse 5′-TGG TCC AGG GTT TCT TAC TCC-3′.

### 2.8. Western Blotting Analysis

Liver tissues were homogenized in radioimmunoprecipitation assay (RIPA) lysis buffer (MultiSciences, Hangzhou, China) containing protease inhibitors (Servicebio, Wuhan, China); and protein quantification was performed using the bicinchoninic acid (BCA) protein assay kit (MultiSciences, Hangzhou, China). Equal amounts of proteins were separated by 10% sodium dodecyl sulfate-polyacrylamide gel electrophoresis (SDS-PAGE) and transferred to polyvinylidene fluoride (PVDF) membranes. After blocking with 5% nonfat milk for 1 hr at room temperature, incubate at 4°C with the following specific antibodies: anti-caspase1 (24232S, CST), anticleaved caspase1 (89332S, CST), anti-ASC (67824S, CST), anti-NLRP3 (15101S, CST), anti-TLR4 (14358S, CST), anti-pNF-*κ*B (3033S, CST), anti-NF-*κ*B (8242S, CST), anti-IL-1*β* (12242S, CST), anti-HMGB1 (6893S, CST), and anti-GAPDH (GB12002-100, Servicebio). All antibodies were diluted 1 : 1,000. After incubation, the PVDF membrane was washed three times with Tween-20 in Tris-HCl buffer for 5 min each. The PVDF membrane was then incubated with horseradish peroxidase (HRP)-conjugated secondary antibody for 1 hr at room temperature. Immunoreactivity was determined using an enhanced chemiluminescence reagent ECL kit (Beyotime, Shanghai, China). Gray values were detected by the ImageJ system (National Institutes of Health). GAPDH was used as the loading control.

### 2.9. Immunofluorescence Analysis

The liver paraffin sections were baked in an oven at 65°C for 30 min, followed by deparaffinization, hydration, and antigen retrieval. The paraffin sections were then treated with PBS containing 1% Triton X-100 for 1 hr and blocked with 5% donkey serum albumin for 1 hr. Finally, the sections were exposed to specific primary antibodies (NLRP3, cleaved caspase-1, HMGB1, and phosphorylated p65) and incubated at 4°C overnight. The sections were washed three times with PBS for 5 min each; then the sections were incubated with a fluorescent secondary antibody (8889S and 4408S, CST) for 1 hr at room temperature in the dark, and then counterstained with DAPI for 5 min. Images were observed using a confocal laser scanning microscope (Leica, Germany).

### 2.10. Statistical Analysis

Data are presented as the mean ± standard deviation (SD). Graphs were generated, and statistical analyses were conducted using GraphPad Prism 7 software (GraphPad Software, San Diego, CA, USA). The results were analyzed using the one-way analysis of variance (ANOVA). A *p*-value of less than 0.05 was considered to indicate statistical significance.

## 3. Results

### 3.1. ALO Pretreatment Attenuates APAP-Induced ALI

**Figure 1:**
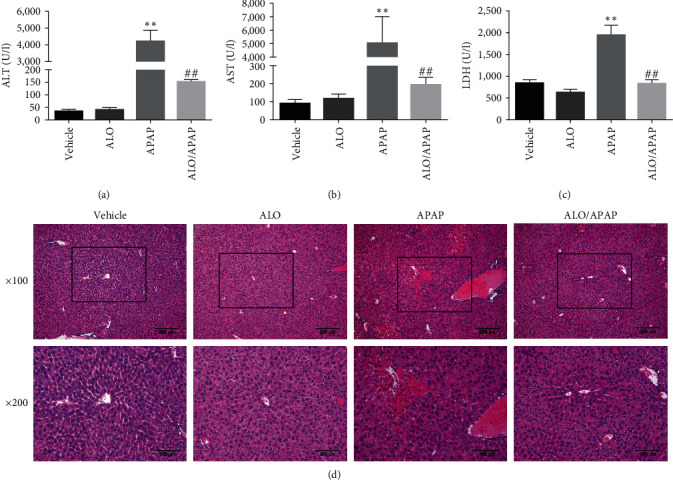
Serum levels of ALT (a), AST (b), and LDH (c), as well as hematoxylin and eosin (H&E) staining (d) were evaluated in mice pretreated with ALO to assess the protective effects against acetaminophen (APAP)-induced acute liver injury. Data are presented as means ± SD (*n* = 6–8 for each group).  ^*∗∗*^*p* < 0.01 versus the vehicle group; ^##^*p* < 0.01 versus the APAP group. Three independent experiments were performed.

To study the effect of ALO on APAP-induced ALI, serum liver function biochemical indicators were detected, and liver pathological sections were assessed by H&E staining. It was found that the serum ALT, AST, and LDH levels of mice in the APAP group were significantly higher than those in the vehicle group (Figures 1(a), 1(b), and 1(c)). As expected, ALO had no effect on the liver function of normal healthy mice; while the ALO/APAP group showed significantly lower levels of ALT, AST, and LDH compared with the APAP group. Pathological changes in liver tissue were examined using light microscopy to determine infiltration of inflammatory cells and necrosis of liver tissue. The results were shown in Figure 1(d), the liver tissues of the vehicle group and ALO group showed normal hepatic lobular structure, and the central vein and hepatic cord were clearly visible. APAP administration resulted in hepatic lobular structural disorder, hepatocyte swelling and rupture, and extensive cell necrosis with inflammatory cell infiltration; ALO pretreatment significantly ameliorated these pathological lesions. These results demonstrated that pretreatment with ALO exerted a significant protective effect against APAP-induced ALI in mice.

### 3.2. ALO Pretreatment Reduced Hepatic Oxidative Stress in APAP-Induced ALI

**Figure 2:**
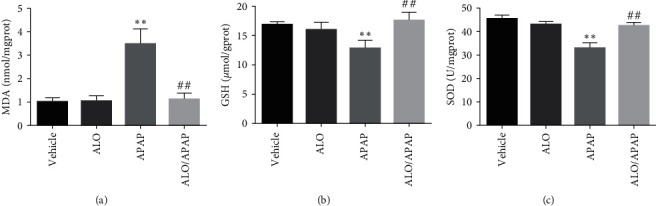
The levels of MDA (a), GSH (b), and SOD (c) in the liver tissues were measured to assess the oxidative stress and antioxidant status in mice pretreated with ALO before inducing acute liver injury with APAP. Data are presented as mean ± SD (*n* = 6–8 for each group).  ^*∗∗*^*p* < 0.01 versus the vehicle group; ^##^*p* < 0.01 versus the APAP group. Three independent experiments were performed.

Given that oxidative stress is a pivotal and causative factor in the pathogenesis of APAP-induced ALI, the hepatic concentrations of MDA and GSH, as well as the activity of SOD, which are key indicators of oxidative stress and antioxidant status, were investigated. MDA, a biomarker of lipid peroxidation, is a critical product of oxidative stress, whereas SOD and GSH are endogenous antioxidants that protect cells from oxidative damage. In this study, when compared to the vehicle group, the levels of MDA, GSH, and SOD activity in the ALO group did not exhibit any significant differences, indicating that ALO alone does not affect these parameters under normal conditions. Contrastingly, the administration of APAP led to a pronounced elevation in hepatic MDA levels (Figure 2(a)), alongside a significant reduction in both GSH content and SOD activity (Figures 2(b) and 2(c)). These alterations indicative of oxidative stress were notably attenuated in the group pretreated with ALO. These results underscore the antioxidant properties of ALO and suggest that pretreatment with ALO can substantially mitigate the oxidative stress associated with APAP-induced ALI.

### 3.3. ALO Pretreatment Inhibited the Expression of Pro-Inflammatory Cytokines in APAP-Induced ALI

**Figure 3:**
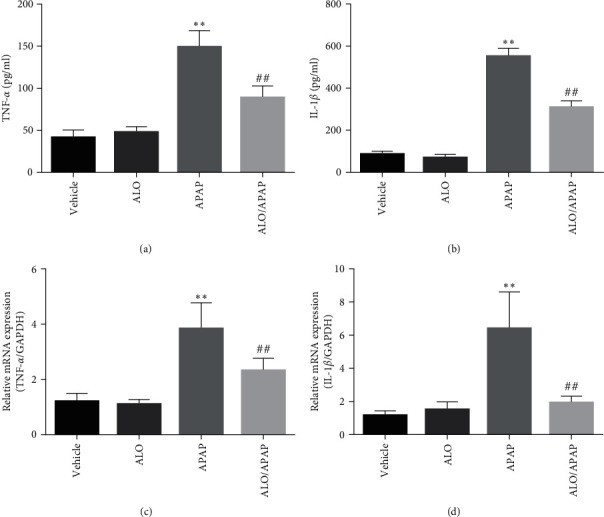
The expression levels of TNF-*α* (a and c) and IL-1*β* (b and d) in both serum and liver tissues of mice pretreated with ALO were measured using ELISA and q-PCR to evaluate the inflammatory response in APAP-induced acute liver injury. Data are presented as mean ± SD (*n* = 6–8 for each group).  ^*∗∗*^*p* < 0.01 versus the vehicle group; ^##^*p* < 0.01 versus the APAP group. Three independent experiments were performed.

Several studies have identified an inflammatory response, characterized by the production of cytokines such as IL-6, TNF-*α*, and IL-1*β*, as a hallmark feature of APAP-induced ALI [25, 27]. To ascertain whether ALO could mitigate this inflammatory response, the expression levels of TNF-*α* and IL-1*β* were measured across different treatment groups. As shown in Figures 3(a) and 3(b), no significant differences were observed between the ALO group and the vehicle group, indicating that ALO does not modulate these cytokines under basal conditions. In contrast, APAP treatment led to markedly elevated levels of TNF-*α* and IL-1*β* when compared to the vehicle group, indicative of an induced inflammatory state. Notably, pretreatment with ALO significantly reduced the expression of these inflammatory cytokines. Subsequently, the mRNA levels of these pro-inflammatory cytokines in liver tissue were assessed. Consistent with the protein expression data, the mRNA levels of TNF-*α* and IL-1*β* were found to be significantly upregulated in the APAP group. Importantly, the ALO/APAP group exhibited significantly reduced mRNA expression levels of these cytokines compared to the APAP group (Figures 3(c) and 3(d)).

### 3.4. ALO Pretreatment Reduced APAP-Induced NLRP3/Inflammasome Activation Pathway

**Figure 4:**
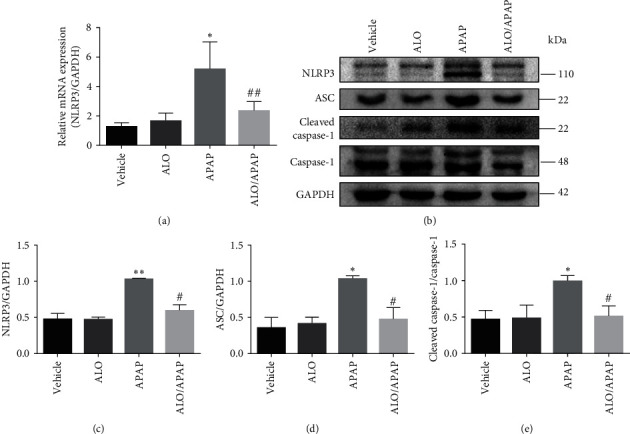
The effects of ALO on NLRP3 inflammasome activation in APAP-induced acute liver injury. (a) Relative mRNA expression of NLRP3 in liver tissue was detected by q-PCR. (b) The protein expressions of NLRP3, ASC, caspase-1, and cleaved caspase-1 were detected by Western blot. (c–e) Quantitative analysis of (b). Results are representative of three independent experiments and data are presented as mean ± SD (*n* = 6–8 for each group).  ^*∗*^*p* < 0.05 and  ^*∗∗*^*p* < 0.01 versus the vehicle group; ^#^*p* < 0.05 and ^##^*p* < 0.01 versus the APAP group.

The NLRP3 inflammasome is recognized for its pivotal role in the pathogenesis and progression of a spectrum of liver diseases [28]. To determine the effect of ALO on NLRP3 inflammasome activation in APAP-induced ALI, q-PCR was utilized to measure the mRNA levels of the inflammasome component NLRP3. The results showed no significant difference in NLRP3 mRNA levels between the ALO group and the vehicle group. However, a significant upregulation of NLRP3 mRNA was observed in the APAP group when compared to the vehicle group, which was notably attenuated by ALO pretreatment (Figure 4(a)).

Simultaneously, the expression levels of the NLRP3 inflammasome complex components, including NLRP3, apoptosis-associated speck-like protein containing a CARD (ASC), and caspase-1, were assessed using Western blot analysis. As shown in Figures 4(b), 4(c), 4(d), and 4(e), there were no significant differences in the protein expression levels of NLRP3, ASC, and the ratio of cleaved caspase-1 to total caspase-1 when the ALO group was compared to the vehicle group. In contrast, APAP administration led to a pronounced increase in the protein expression of these inflammasome components, which was significantly mitigated by ALO pretreatment.

**Figure 5:**
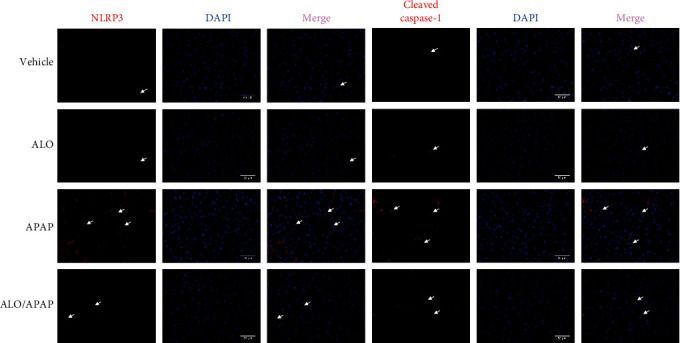
The expression of NLRP3 and cleaved caspase-1 in APAP-induced acute liver injury by immunofluorescence analysis (original magnification, ×400). Three independent experiments were performed.

Subsequently, the expression and cellular localization of NLRP3 and cleaved caspase-1 in the context of APAP-induced ALI were examined through immunofluorescence staining. High levels of cytoplasmic NLRP3 and cleaved caspase-1 were observed in the APAP group, as shown in Figure 5, whereas ALO pretreatment markedly suppressed their expression. Notably, ALO treatment alone did not exert any effect on these parameters.

Therefore, these findings collectively demonstrate that ALO pretreatment can significantly suppress the activation of the NLRP3 inflammasome in APAP-induced ALI.

### 3.5. ALO Pretreatment Inhibited the Activation of HMGB1/TLR4/NF-*κ*B Signaling Pathway in APAP-Induced ALI

**Figure 6:**
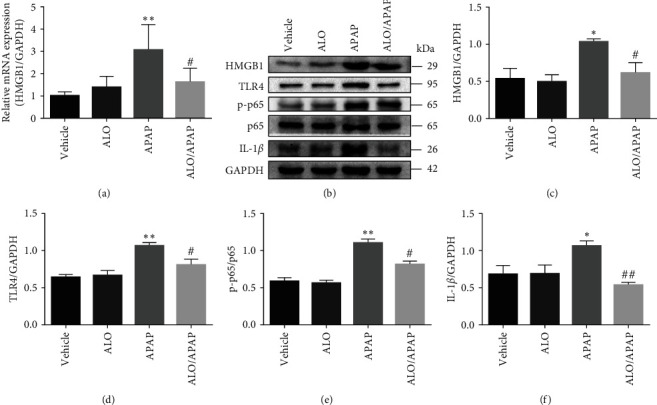
The effects of ALO pretreatment on the HMGB1/TLR4/NF-*κ*B signaling pathway during APAP-induced acute liver injury. (a) The relative mRNA expression of HMGB1 in liver tissue was detected by q-PCR. (b) The expressions of HMGB1, TLR4, p65, p-p65, and IL-1*β* proteins were detected by Western blot. (c–f) Quantitative analysis of (b). Results are representative of three independent experiments and data are presented as mean ± SD (*n* = 6–8 for each group).  ^*∗*^*p* < 0.05 and  ^*∗∗*^*p* < 0.01 versus the vehicle group; ^#^*p* < 0.05 and ^##^*p* < 0.01 versus the APAP group.

HMGB1 plays a crucial role in the pathogenesis of various liver injury diseases, known to initiate inflammatory responses by upregulating the expression of pro-inflammatory cytokines, such as TNF-*α* and IL-1*β*, through the NF-*κ*B signaling pathway [28, 29]. To investigate the potential of ALO to protect against APAP-induced ALI in mice through the inhibition of the HMGB1/TLR4/NF-*κ*B signaling pathway, both the HMGB1 mRNA expression levels and the activation status of the HMGB1/TLR4/NF-*κ*B signaling pathway were detected.  Figure 6(a) illustrates a significant increase in HMGB1 mRNA levels in the APAP group compared to the vehicle group, with a notable reduction in the ALO pretreatment group. ALO treatment alone did not affect the HMGB1 mRNA levels. Furthermore, APAP administration led to a significant increase in the protein expressions of HMGB1, TLR4, phosphorylated p65 (p-p65), and IL-1*β*, as compared to the vehicle group. However, ALO pretreatment effectively mitigated the expression of these proteins, as shown in Figures 6(b), 6(c), 6(d), 6(e), and 6(f). Notably, ALO alone did not exert any effect on these parameters.

**Figure 7:**
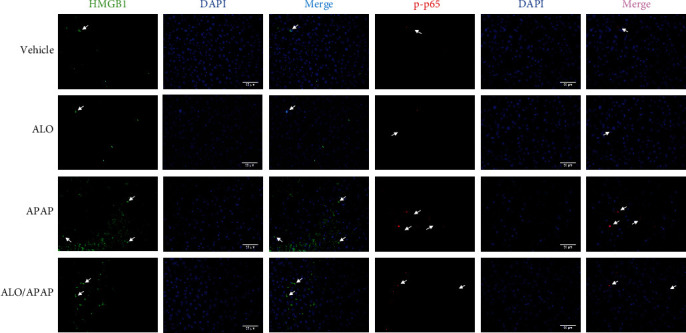
The expression of HMGB1 and p-p65 in APAP-induced acute liver injury by immunofluorescence analysis (original magnification, ×400). Three independent experiments were performed.

Additionally, the immunofluorescence analysis was employed to detect the expression and cellular localization of HMGB1 and p-p65 in the context of APAP-induced ALI. Elevated cytoplasmic HMGB1 and p-p65 expression levels were observed in the APAP group, indicative of an active inflammatory response. Importantly, ALO pretreatment significantly reversed these increases, as depicted in Figure 7. ALO treatment alone, however, showed no effect on the expression and localization of these proteins. These findings collectively suggest that the hepatoprotective effect of ALO in APAP-induced ALI is closely associated with the inhibition of the HMGB1/TLR4/NF-*κ*B signaling pathway activation, thereby potentially attenuating the inflammatory process and liver damage.

## 4. Discussion

DILI is a prevalent clinical adverse drug reaction, encompassing liver damage caused by chemical medications, Chinese herbal medicines, dietary supplements, and their metabolites. DILI is a primary cause of ALI, with severe cases potentially leading to ALF and patient mortality [29]. Clinical surveys have indicated a rising incidence of DILI, now ranking as the third most common cause of liver disease, after viral hepatitis and fatty liver disease [30]. Although APAP is a safe and efficacious antipyretic and analgesic at recommended doses, overdose can result in ALI due to its hepatotoxicity. While NAC serves as the primary antidote for APAP poisoning, its effectiveness is limited to the early stages of APAP-induced ALI. Hence, there is an urgent need to identify novel therapeutic targets and develop efficacious treatments for ALI. Previous research has shown that ALO possesses therapeutic effects in various inflammatory disease models [31]. However, its potential benefits in APAP-induced ALI were previously unknown. This study, therefore, explores the protective effects and underlying mechanisms of ALO on APAP-induced ALI.

APAP-induced hepatotoxicity initiates with the formation and accumulation of the toxic metabolite NAPQI, which binds to intracellular proteins, triggering oxidative stress, mitochondrial dysfunction, ATP depletion, and hepatocyte necrosis [32]. In this study, an ALI model was established by intraperitoneally injecting APAP (150 mg/kg) into mice. H&E staining revealed tissue hemorrhage, edema, and inflammatory cell infiltration in the livers of APAP-treated mice. Additionally, serum levels of ALT, AST, and LDH were significantly elevated, alongside increased oxidative stress and pro-inflammatory cytokines (TNF-*α* and IL-1*β*). ALO pretreatment, however, significantly mitigated these pathological changes and decreased the associated indices. Furthermore, the therapeutic effects of ALO on APAP-induced ALI were evaluated, with results indicating similar benefits to preventive administration across histopathological examination, liver function indicators, and inflammatory cytokines. These findings underscore the protective role of ALO in APAP-induced ALI. Given that higher doses of APAP (300 mg/kg) have been reported to establish ALI models [33], the protective efficacy of ALO at such doses remains to be determined. Demonstrating ALO's therapeutic potential in high-dose APAP-induced ALI could position it as a valuable adjunct to NAC in clinical ALI treatment. Additionally, assessing ALO's toxicity is essential and should be addressed in future research.

An inflammatory response is a hallmark of ALI pathology and often accompanies its pathological progression [34, 35]. In this study, the expression of pro-inflammatory cytokines TNF-*α* and IL-1*β* was significantly upregulated in APAP-induced ALI mouse models and was substantially reduced by ALO pretreatment. The NLRP3 inflammasome signaling pathway is integral to liver disease pathology [28]. Upon activation by cellular stress signals, NLRP3 binds to ASC and procaspase-1, forming the NLRP3 inflammasome complex. This complex cleaves procaspase-1 into its active form through autocatalysis. Activated caspase-1, in turn, cleaves pro-IL-1*β* and pro-IL-18 into their mature forms, IL-1*β* and IL-18, respectively [36, 37, 38]. Concurrently, activated caspase-1 also processes Gasdermin D (GSDMD) into a N-terminal fragment (GSDMD-N). GSDMD-N forms a transmembrane pore that releases cytokines such as IL-1*β* and IL-18, leading to robust inflammation and cell death [39]. Recent reports indicate that levels of NLRP3 and cleaved caspase-1 are significantly elevated in APAP-induced rat hepatocytes, demonstrating the involvement of inflammasome activation in APAP-induced hepatocyte death [16]. In the present study, the expression of NLRP3, ASC, and cleaved caspase-1 was notably increased in the APAP-induced ALI mouse model. Notably, ALO pretreatment was found to ameliorate pathological changes by inhibiting NLRP3 activation and the associated inflammatory response.

HMGB1 is a pro-inflammatory cytokine released by cells in response to stimulation or injury [40]. Additionally, HMGB1 functions as a damage-associated molecular pattern (DAMP) molecule, contributing to the activation of the NLRP3 inflammasome [41]. The previous research identified a significant upregulation of HMGB1 expression in liver tissue within a mouse model of APAP-induced ALI [25]. Moreover, HMGB1 has been shown to mediate NLRP3 inflammasome activation in ALI models induced by hemorrhagic shock and heat shock [42, 43]. Previous studies have demonstrated that HMGB1 initiates the innate immune response and promotes inflammation through the TLR4/NF-*κ*B signaling pathway, which can exacerbate liver injury [44]. In the context of acute glaucoma, HMGB1 also enhances the expression of NLRP3 and caspase-8 by activating the NF-*κ*B pathway [45]. Consequently, NF-*κ*B plays a crucial role in the expression of the NLRP3 inflammasome and its substrates, IL-1*β*, and IL-18 [46]. In the current study, the expression levels of HMGB1, TLR4, NF-*κ*B, and NLRP3 were found to be upregulated in the APAP-induced ALI model. Notably, pretreatment with ALO significantly inhibited the activation of NF-*κ*B and the release of HMGB1. It is proposed that the protective mechanism of ALO against APAP-induced ALI may involve the inhibition of the HMGB1/TLR4/NF-*κ*B and NLRP3/inflammasome signaling pathways, thereby reducing the release of pro-inflammatory cytokines.

## 5. Conclusions

The collective findings suggest that ALO has the potential to ameliorate APAP-induced ALI through the inhibition of key inflammatory pathways, namely the HMGB1/TLR4/NF-*κ*B and NLRP3 inflammasome pathways. This inhibition leads to a reduction in the release of pro-inflammatory cytokines and an alleviation of APAP-induced hepatic oxidative stress. Despite these advancements, our current understanding acknowledges that the protective effects of ALO may extend beyond the mere suppression of inflammatory responses. It is plausible that ALO engages a spectrum of noninflammatory protective mechanisms that have yet to be fully elucidated and thus merit further investigation. In conclusion, these findings collectively underscore the complexity of ALI pathophysiology and highlight the multifaceted nature of ALO's protective mechanisms, which could offer promising directions for future hepatoprotective treatments.

## Data Availability

All data and figures used to support the findings of this study are included within the article.

## Abbreviations

ALF: Acute liver failure
ALI: Acute liver injury
ALT: Alanine aminotransferase
ALO: Aloperine
APAP: N-acetyl-para-aminophenol
ASC: Apoptosis-associated speck-like protein containing a CARD
AST: Aspartate aminotransferase
BCA: Bicinchoninic acid
DAMP: Damage-associated molecular pattern
DILI: Drug-induced liver injury
GAPDH: Glyceraldehyde-3-phosphate dehydrogenase
GSDMD: Gasdermin D
GSDMD-N: N-terminal fragment of GSDMD
GSH: Glutathione
H&E: Hematoxylin–eosin
HMGB1: High-mobility group box 1
HRP: Horseradish peroxidase
LDH: Lactate dehydrogenase
LPS: Lipopolysaccharide
MDA: Malondialdehyde
NAC: N-acetylcysteine
NAPQI: N-acetyl-p-benzo-quinone imine
NF-*κ*B: Nuclear factor-kappa B
OVA: Ovalbumin
PBS: Phosphate-buffered saline
PVDF: Polyvinylidene fluoride
q-PCR: Quantitative real-time polymerase chain reaction
RIPA: Radioimmunoprecipitation assay
ROS: Reactive oxygen species
SDS-PAGE: Sodium dodecyl sulfate-polyacrylamide gel electrophoresis
SOD: Superoxide dismutase.
